# 肺癌循环肿瘤细胞侵袭转移机制的研究进展

**DOI:** 10.3779/j.issn.1009-3419.2020.03.09

**Published:** 2020-03-20

**Authors:** 春国 茆, 波 邓

**Affiliations:** 400042 重庆，陆军军医大学大坪医院全军胸外科研究所 Department of Thoracic Surgery, Daping Hospital, Army Medical University, Chongqing 400042, China

**Keywords:** 肺肿瘤, 循环肿瘤细胞, 机制, 分子标志物, 免疫逃逸, 检测方法, 临床应用, Lung neoplasms, Circulating tumor cells, Generative mechanism, Molecular maker, Immune escape, Detection method, Clinical application

## Abstract

近年来，随着液体活检技术兴起，循环肿瘤细胞（circulating tumor cell, CTC）在癌症患者的早期诊断、疗效评估及预后评价等方面显示出重要的价值。CTC的产生导致肿瘤发生远处转移及患者的预后不良。因此，阐明CTC的产生、进入循环系统及其免疫逃逸的机制尤为重要。目前，精准诊疗成为提高肺癌患者预后的重要努力方向。针对肺癌CTC有望为肺癌精准诊疗提供有力的理论依据与重要手段。现对上述热点问题的最新研究进展进行综述。

## Abstract (en)

In recent years, with the emergence of liquid biopsies, circulating tumor cells (CTCs) demonstrated significant value in the early diagnosis of patients with cancer, curative effect evaluation and prognosis evaluation. CTCs are the main cause of tumor metastasis, which indicates the poor prognosis of patients. Therefore, it is particularly important to elucidate the mechanism of CTC's generation, how to entry into circulatory system and immune escape. At present, accurate diagnosis and treatment has become an important direction to improve the prognosis of lung cancer patients. CTC for lung cancer is expected to provide a strong theoretical basis and important methods for accurate diagnosis and treatment of lung cancer. This paper reviews the latest research progress on the above hot issues.

癌症是人类健康的一大威胁，其发病率和死亡率在全球范围内迅速增长，2018年全世界新增1,810万新发病例和960万癌症死亡病例，而肺癌的发病率及死亡率居各种癌症之首^[1]^。肺癌作为第一大癌症杀手，发病隐匿、诊断不及时、易转移复发。1869年澳大利亚医师Ashworth首次提出循环肿瘤细胞（circulating tumor cell, CTC）的概念，即：CTC是指原发肿瘤或转移灶脱落后释放入外周血中随血液循环的肿瘤细胞。随着检测手段和技术方法的不断进步，CTC检测与研究逐渐成为当前肿瘤研究的热点和焦点。理论上，外周血中CTC的出现是肿瘤远处转移的前提，是形成转移灶的关键环节^[2]^。因此，CTC检测对肺癌的早期诊断、临床分期、治疗及预后评估具有重要的临床意义^[2]^。肺癌CTC如何脱落产生、进入循环、发生免疫逃避及避免凋亡，并在新的微环境下成瘤？本文拟对上述焦点问题进行综述。

## 1 CTC的产生机制

### 1.1 CTC从肿瘤组织中脱落的机制

CTC从肿瘤组织中脱落与肿瘤上皮-间质转化（epithelial-mesenchymal transition, EMT）有关^[3]^。肿瘤细胞在EMT过程中，下调表达上皮标志物（如上皮细胞黏附分子、细胞角蛋白和E-钙黏蛋白），上调表达间质标志物（如波形蛋白、N-钙黏蛋白等），从而获得某些干细胞特征并增强自身侵袭力^[4]^。所以，肺癌患者血液中不仅存在上皮细胞表型的CTC，还存在间质细胞表型及上皮间质混合表型的CTC^[5]^。此外，EMT还与肿瘤微环境有关，EMT后基质金属蛋白酶在细胞外基质中表达上调可引起基质降解，从而使肿瘤细胞易于从肿瘤组织中脱落下来，形成CTC，导致肿瘤的转移复发^[6]^。

### 1.2 CTC进入外周毛细血管（破壁）机制

CTC进入外周毛细血管的机制复杂，肿瘤自身分泌细胞因子在调控其血管内渗特别是形成侵袭性伪足方面发挥重要作用。例如Wiskott-Aldrich综合征蛋白家族WAS-like蛋白对肌动蛋白细胞骨架重塑的调节有助于肿瘤细胞的血管内渗^[7]^。肿瘤微环境及肿瘤血管生成亦有助于CTC的产生。肿瘤细胞可诱导细胞外基质成分的改变从而帮助其向血管渗入^[8]^。转化生长因子β（transforming growth factor-beta, TGF-β）可通过诱导肿瘤细胞EMT使肿瘤细胞渗入血管内，而阻断TGF-β可抑制肿瘤细胞的存活、迁移和转移。另外，TGF-β还可激活激酶TAK1，调控血管内皮细胞坏死凋亡，从而帮助肿瘤细胞进入血管^[9]^。

### 1.3 CTC在外周血中避免失巢凋亡、避免免疫杀伤的机制

当肿瘤细胞进入外周血液中后，其受到机体免疫细胞的攻击、血液流体剪切力的影响以及失去细胞外基质的营养和黏附作用，最终导致CTC大量凋亡。鉴于CTC抗失巢凋亡能力较弱，因此CTC“抱团”形成CTC簇，有助于帮助其逃避失巢凋亡，研究^[10]^证实，CTC簇的形成及集体迁移还可提高肿瘤细胞的转移能力。在抗肿瘤免疫应答中，细胞程序性死亡配体1（programmed cell death 1 ligand 1, PD-L1）在肿瘤细胞的表达使得T淋巴细胞不能有效杀伤CTC而发生免疫逃逸^[11]^；肿瘤细胞表达PD-L1，低表达或不表达CD80和CD86这类活化T淋巴细胞的共刺激分子，使其不能为T细胞的活化提供第二信号，肿瘤细胞产生免疫耐受。此外，肿瘤患者血液中不同细胞成分在促进CTC的免疫逃逸方面也起重要作用。血小板与肿瘤细胞之间的相互作用在肿瘤细胞逃避免疫监视上发挥着重要的作用，血小板附着于CTC表面，以致肿瘤浸润淋巴细胞不能有效杀灭CTC^[12]^。肿瘤细胞诱导的调节性T细胞与髓源性抑制细胞也具有免疫抑制作用，是免疫治疗的主要障碍^[13]^。因此，深入研究CTC有助于促进肿瘤免疫治疗的深入变革，引领肿瘤治疗的新策略。

### 1.4 CTC促进肿瘤新生血管生成的机制

肿瘤新生血管生成是CTC生长和转移形成的必要条件^[11]^。其中，血管内皮细胞生长因子（vascular endothelial growth factor, VEGF）是肿瘤血管生成的最重要的刺激因子。有研究^[14]^显示VEGF与CTC均升高的患者其生存期明显缩短。而血管生成素（angiopoietin）在促进肿瘤血管生成上也发挥着重要的调节作用，血管生成素2与VEGF结合，可促进新血管形成^[15]^。此外，基质金属蛋白酶高表达可调节细胞外基质结构从而促进肿瘤新生血管生成^[16]^。进一步的研究^[17]^证实，CTC还可发生肿瘤原位播种，通过电子数字成像技术对动物模型的染色血管成像显示，侵袭性的CTC能促进肿瘤血管的生成，肿瘤血管的平均长度及分支增加。

## 2 CTC的捕获方法

目前，CTC的捕获方法主要分为两类：基于CTC本身的物理特性，即CTC的大小、密度、比重、电荷、离心力等，例如，经典的滤过膜法、蛋白电泳、密度梯度法和微流芯片法；基于CTC免疫学特性，即肿瘤细胞表面的特异性抗原表达（阳性富集）或正常血细胞标志物（阴性富集）。

### 2.1 基于物理特性的捕获方法

#### 2.1.1 膜过滤法

利用CTC与正常血细胞的体积差异（CTC较正常血细胞体积大），通过滤过膜将CTC与血细胞分离。优点：不依赖CTC的分子标志物，可分离不同表型的CTC并进行细胞形态学、免疫细胞学及遗传学检测；缺点：体积小的肿瘤细胞可能被过滤而逃脱分离。但有研究^[17]^使用此法进行CTC捕获，证实91%的乳腺癌CTC可被捕获，分离效率令人满意。

#### 2.1.2 电泳法

电泳法是利用细胞极性的一种捕获方法。利用肿瘤细胞的固有特性，无需标记肿瘤细胞，且此方法还可提供未经修饰的、活肿瘤细胞用于培养以及后续分析。有研究^[18]^将双向蛋白电泳与阻抗测量相结合的技术对CTC进行捕获与检测，这种简单、快速、无标签、低成本的方法可能为开发未来应用的细胞诊断系统提供新方法。

#### 2.1.3 密度梯度离心法

根据肿瘤细胞与血细胞的不同密度的物理特性，通过密度梯度离心进行细胞的分离，从而富集出所需CTC。一项研究^[19]^显示，此方法可在每毫升血液中检出6个-10个CTC。另外，有研究^[20]^将离心微流装置与新型的流体辅助技术相结合用于CTC的检测，其敏感性与特异性分别高达85.3%、90.3%。

### 2.2 基于免疫学特性的捕获方法

利用CTC分子抗原与免疫磁珠抗体结合再进行磁性分离。该法优点：特异性、选择性、灵敏性高，不影响细胞活性等；缺点：利用单一免疫方法容易出现漏检，不能获得全部CTC信息。随着科技进步，新的技术正逐步被开发利用。例如，将抗上皮细胞黏附分子抗体修饰在磁性纳米簇上直接对CTC进行分选就是一种高效能阳性捕获方法，此方法不仅对CTC的结合表现出良好的亲和性，而且还能有效地富集CTC^[21]^。此外，亦有依靠上皮其他分子标志物如细胞角蛋白或波形蛋白的捕获方法^[22]^。

## 3 CTC的鉴别方法

由于CTC在外周血循环中的数量极少，我们在捕获这些细胞的同时，更要对这些细胞进行鉴别分选，证实确为CTC，然后才能进一步对其研究分析。

### 3.1 CTC的分子标志物

#### 3.1.1 上皮细胞分子标志物

如上皮细胞黏附分子（epithelial cell adhesion molecule, Ep-CAM）等较早应用于CTC鉴定。例如，Cell Search^®^、MACS、CTC芯片等方法均通过富集表达Ep-CAM的细胞，来捕获CTC^[23]^。但往往需要结合间质型标志物或其他分子标志物。

##### 3.1.1.1 Ep-CAM

Ep-CAM是一种跨膜糖蛋白，在上皮来源的肿瘤中普遍表达，与细胞黏附、迁移、增殖以及肿瘤的发生、发展有关。但仅依靠单一上皮细胞分子标志物对CTC进行鉴定不全面。EMT过程会导致CTC出现不同表型的亚群：即上皮细胞型、间质型以及两者兼有的混合型。基于Ep-CAM研发的CellSearch检测系统对CTC的检测灵敏性仅为32%-70%。因此，需对CTC的分子标志物特征进行多方面的研究，以便得到更精确的结论。

##### 3.1.1.2 细胞角蛋白（cytokeratin, CK）

CK主要分布于上皮细胞，是主要的细胞骨架蛋白，具有维持上皮组织的完整性及连续性等重要功能，并与上皮细胞的增殖分化密切有关。现已成为常用的肿瘤免疫组织化学标记物，并用于癌症的诊断、治疗及预后评价^[24]^。一项基于66例小细胞肺癌患者的研究^[25]^采用抗CK、抗CD45以及抗波形蛋白的抗体相结合的方法对CTC进行富集分选，发现CTCs在小细胞肺癌患者中具有重要的表型异质性。另有研究^[26]^证实，在162个乳腺癌转移的淋巴结样本中155例可表达CK，推断可依据CK分析治疗前后前哨淋巴结的肿瘤浸润情况。

##### 3.1.1.3 E-钙黏蛋白（E-cadherin）

E-cadherin是钙黏蛋白家族中的一类成员，主要在上皮组织中表达。E-cadherin缺失与EMT发生及癌症的发生与转移有关。E-cadherin可帮助癌细胞穿过细胞基底膜侵入周围组织。E-cadherin亦可作为CTC辅助分子标志物。有研究^[27]^表明，EMT过程导致肿瘤细胞丢失E-cadherin，这也是一线化疗耐药的重要原因。而且，亦有研究^[28]^将E-cadherin与Ep-CAM、CK等联合作为CTC的分子标志物。

#### 3.1.2 间质细胞分子标志物

相比仅有上皮表型的CTC，具有间质表型的CTC由于发生过EMT，具有更高的侵袭转移能力。因此，同时利用上皮及间质细胞分子标志物对CTC进行分类及鉴定有助于分辨出高侵袭性CTC亚群^[22]^。

##### 3.1.2.1 波形蛋白（Vimentin）

Vimentin为中间丝蛋白，主要在间充质细胞中表达，具有维持细胞完整性的重要作用。在恶性肿瘤患者化疗效果评价中，Vimentin与Ep-CAM可作为上皮型与间质型CTC的分类依据^[29]^，研究^[30]^证实，使用CellSearch技术联合检测细胞表面Vimentin阳性CTC有助于评估化疗反应并为乳腺癌患者的个体化治疗提供新思路。监测Vimentin阳性CTC可应用于肿瘤患者的复发预警与生存评估。在胰腺癌复发患者中，Vimentin阳性和细胞角蛋白表达CTCs的患者比没有CTCs的患者的中位复发时间减少^[31]^。

##### 3.1.2.2 N-钙黏蛋白（N-cadherin）

N-cadherin是一种钙依赖跨膜糖蛋白，参与调节钙介导的细胞黏附、迁移与维持细胞形态。癌细胞通过EMT过程，高表达N-cadherin，与肿瘤的侵袭转移有关，并与肿瘤治疗获得性耐药相关^[32]^。通过联合检测Ep-CAM与N-cadherin可以提高卵巢癌CTC的检出率^[33]^。另外，一项*meta*分析^[34]^显示，N-钙黏蛋白表达上调与上皮性实体瘤预后不良有关。

##### 3.1.2.3 其他新兴分子标志物

###### 3.1.2.3.1 乳腺癌抗雌激素耐药蛋白1（breast cancer anti-estrogen resistance protein 1, BCAR1）

BCAR1又名p130cas，相对分子质量为130 kDa，能与包括Crk、src和FAK在内的多种蛋白质结合并发生相互作用。研究^[35, 36]^表明，BCAR1与肿瘤的侵袭、转移、增殖、分化、存活等密切相关，有望成为新的CTC标志物，监测BCAR1阳性CTC有助于预测早期肺腺癌复发或转移^[37]^。

###### 3.1.2.3.2 PD-L1

PD-L1是在肿瘤中表达的，大小为40 kDa的I型跨膜蛋白，其与T细胞表面的PD-1结合，可以帮助肿瘤细胞发生免疫逃避。研究^[38]^表明，PD-L1表达可以作为Pembrolizumab治疗晚期非小细胞肺癌的疗效预测生物标志物，一项关于肺癌CTC的研究^[39]^发现，CTC中PD-L1阳性表达为50.5%-67.0%，并与癌组织中PD-L1表达密切相关。而CTC中PD-L1的高表达与非小细胞肺癌患者预后不良有关^[40]^。推测PD-L1阳性CTC可能通过诱导免疫逃避，导致肿瘤侵袭转移^[41]^。

###### 3.1.2.3.3 叶酸受体（folate receptor, FR）

FR是一种糖基化磷脂酰肌醇偶联蛋白，对其天然配体叶酸有着较高的亲和力和内吞转运能力，它是一种细胞表面蛋白，已被证实是一个理想的肿瘤标示物。在部分肺癌患者中，FR高表达，而在正常组织低表达或不表达^[42]^。研究表明，FR阳性的CTC不仅可作为非小细胞肺癌新的诊断标志物^[43]^，而且还可以用于小细胞肺癌患者一线化疗的预后评估^[44]^。FR阳性CTC被证实是一个有效的老年人群癌症的早期诊断标志物^[45]^。

### 3.2 鉴定方法

#### 3.2.1 免疫组织化学法

该方法通过抗原抗体免疫反应，并与组织化学技术相结合，通常需与其他方法联合应用才能满足鉴定需要。而且，在基础研究中，可利用该法进行单个细胞的形态验证。例如，利用滤膜捕获细胞后，可通过该法进行细胞形态学和免疫细胞化学分析，以进行表型鉴定和分类^[46, 47]^。

#### 3.2.2 流式细胞术检测法

该法依赖细胞的光散射特性，利用抗原-抗体结合的原理，将荧光物质与肿瘤细胞单抗相结合并使其染色，然后通过荧光抗原抗体检测技术对细胞表面抗原分析，进行细胞分类和亚群分析。研究^[48]^表明，成像流式细胞术可在体内可视化观察血液循环中CTC和树突状细胞的相互作用，利用这一技术，单个CTC和CTC簇的形态很容易被区分。而且，体内荧光流式细胞术可应用于凋亡CTC的检测^[49]^。随着技术的发展，流式细胞术的应用越来越广泛，而且提供了一种方便快捷的检测方法。

#### 3.2.3 聚合酶链反应（polymerase chain reaction, PCR）

以及逆转录聚合酶链反应（reverse transcription-polymerase chain reaction, RT-PCR）包括对DNA进行PCR以及对mRNA进行RT-PCR两种方法。有研究^[43]^指出，利用PCR检测技术对FR阳性CTC的检测可作为非小细胞肺癌的诊断标志物。但一般来说，RT-PCR在CTC的检测上更常用，即将CTC中特异mRNA序列逆转录为DNA片段并扩增，在外周血中检测到特异mRNA序列就提示CTC的存在。该方法的敏感度高、可重复操作，是目前检测CTC最有效的方法。例如，利用RT-PCR可以实时定量检测乳腺癌患者外周血中的细胞CK蛋白等标志物，从而鉴定CTC^[50]^。亦有相关研究^[51]^通过对CK-19进行实时逆RT-PCR检测，可以进行乳腺癌患者外周血CTC的定量，还可评价CTC的预后。

#### 3.2.4 CTC单细胞测序

单细胞测序是对单个细胞进行全基因组或转录组的检测，以确定其基因表达情况。众所周知，同一患者中癌细胞存在异质性。因此，对CTC进行单细胞测序能鉴定单个细胞的基因结构和基因表达状态，反映CTC细胞间的异质性。最新针对非小细胞肺癌间变性淋巴瘤激酶（anaplasticlymphoma kinase, *ALK*）重排耐药机制的研究^[52]^正是应用单细胞测序技术对CTC进行检测，从而阐明*ALK*重排耐药的原因。亦有研究^[53]^使用CellSearch技术捕获CTC，再通过单细胞测序技术监测CTC突变与拷贝数变化来推断小细胞肺癌的演变与进展，其不同临床亚型拷贝数变化阳性预测与阴性预测分别为80.0%、93.7%。

## 4 CTC在肺癌领域的临床应用

目前，在肺癌诊疗中，检测CTC的临床价值成为研究热点，CTC计数与表型分析在肺癌早期诊断、疗效评价、预后预测以及个体化治疗等方面有重要价值，潜在的临床应用前景十分可观。

### 4.1 CTC与肺癌的早期诊断

CTC在肺癌早期诊断方面有较大的应用潜力。研究^[54]^显示，CTC可作为包括肺癌在内的多种癌症的诊断标志物。CTC的检测可作为监测孤立性肺结节和诊断筛查早期肺癌的生物标志物，联合检测CTC和CEA可显著提高肺腺癌的早期检出率（灵敏度为70.2%、敏感度为79.3%），有助于肺腺癌的早期诊断^[55]^。

### 4.2 CTC与肺癌的疗效评价

监测CTC目前被应用于肺癌患者的疗效评价，并获得了初步的结果。一项前瞻队列研究^[56]^显示，CTC检测可作为非小细胞肺癌患者放疗后的疗效评价手段：放疗后外周血CTC阴性的患者肿瘤无复发或进展，而外周血CTC阳性的患者出现肿瘤的进展且中位生存期显著缩短。而且，CTC检测可有效监测早期肺腺癌术后的复发及转移，并可作为术后辅助治疗疗效评价的重要手段^[57]^。

### 4.3 CTC与肺癌的预后预测

CTC亦有望成为肿瘤的预后预测的重要标志物。研究^[58]^显示，小细胞肺癌在放化疗过程，外周血CTC中PD-L1高表达提示预后不良。而一项93例非小细胞肺癌单中心前瞻性研究^[59]^表明，术前CTC检出数目少的患者较CTC检出数目多的患者生存时间更长，无进展时间更长。我们可以推测，某些治疗前CTC处于较高基线的患者，可判定为复发转移高危人群，而加强治疗后有望改善该人群的预后。

在个体化治疗与精准医疗的背景下，肿瘤具有异质性且需动态实时监控，以便更好地指导治疗及预测预后。CTC研究方兴未艾，具有广阔的应用前景，有望为制定肺癌个体化精准治疗策略提供理论依据。当然，CTC研究领域尚有不少问题函待解决，如CTC免疫逃避机制研究，捕获方法改良优化，鉴定手段及表面特异性标志物，尚待进一步深入研究。
